# Uncovering the intricacies of microbial community dynamics at Helgoland Roads at the end of a spring bloom using automated sampling and 18S meta-barcoding

**DOI:** 10.1371/journal.pone.0233921

**Published:** 2020-06-22

**Authors:** Katja Metfies, Johanna Hessel, Robin Klenk, Wilhelm Petersen, Karen Helen Wiltshire, Alexandra Kraberg

**Affiliations:** 1 Helmholtz Young Investigators Group PLANKTOSENS, Alfred Wegener Institute Helmholtz Centre for Polar and Marine Research, Bremerhaven, Germany; 2 Helmholtz Institute for Functional Marine Biodiversity at the University of Oldenburg (HIFMB), Oldenburg, Germany; 3 Biologische Anstalt Helgoland, Shelf Sea System Ecology, Alfred Wegener Institute Helmholtz Centre for Polar and Marine Research, Helgoland, Germany; 4 Institute of Coastal Research, Helmholtz Zentrum Geesthacht Centre for Materials and Coastal Research, Geesthacht, Germany; 5 Biologische Anstalt Helgoland Coastal Ecology, Alfred Wegener Institute Helmholtz Centre for Polar and Marine Research, List, Germany; IRIG-CEA Grenoble, FRANCE

## Abstract

In May 2016, the remote-controlled **Auto**mated **Fi**ltration System for **M**arine Microbes (AUTOFIM) was implemented in parallel to the Long Term Ecological Research (LTER) observatory Helgoland Roads in the German Bight. We collected samples for characterization of dynamics within the eukaryotic microbial communities at the end of a phytoplankton bloom via 18S meta-barcoding. Understanding consequences of environmental change for key marine ecosystem processes, such as phytoplankton bloom dynamics requires information on biodiversity and species occurrences with adequate temporal and taxonomic resolution via time series observations. Sampling automation and molecular high throughput methods can serve these needs by improving the resolution of current conventional marine time series observations. A technical evaluation based on an investigation of eukaryotic microbes using the partial 18S rRNA gene suggests that automated filtration with the AUTOFIM device and preservation of the plankton samples leads to highly similar 18S community profiles, compared to manual filtration and snap freezing. The molecular data were correlated with conventional microscopic counts. Overall, we observed substantial change in the eukaryotic microbial community structure during the observation period. A simultaneous decline of diatom and ciliate sequences succeeded a peak of *Miracula helgolandica*, suggesting a potential impact of these oomycete parasites on diatom bloom dynamics and phenology in the North Sea. As oomycetes are not routinely counted at Helgoland Roads LTER, our findings illustrate the benefits of combining automated filtration with metabarcodingto augment classical time series observations, particularly for taxa currently neglected due to methodological constraints.

## Introduction

Annually recurring spring phytoplankton blooms with high net primary production characterize coastal seas such as the North Sea. This productivity is fundamental for the associated marine ecosystems and biogeochemical cycles [1]. A number of environmental parameters drive the dynamics of plankton communities and phytoplankton blooms. These involve hydrographic and physical parameters, or nutrient availability, and interactions between planktonic organisms such as grazing or parasitism. Consequently, environmental shifts related to climate can be expected to significantly impact plankton composition, and bloom dynamics [2;3]. First experiments suggest that warming might accelerate termination of phytoplankton spring blooms by fungal parasites [4]. Long-term investigations of the composition of the marine microbial communities are a valuable approach for the overall understanding of impacts of environmental change on plankton communities and related marine ecosystem processes. Such data collections are ideal for illustration of patterns in phytoplankton bloom dynamics and for the elucidation of the underlying mechanisms such as environmental forcing or nutrient availability also at historical time scales. Several oceanographic monitoring programs and observation strategies exist which can provide interdisciplinary insights into processes and dynamics of the marine ecosystem, covering different research areas worldwide, including North Atlantic seas [2;3;5]. The Alfred Wegener Institute, Helmholtz Centre for Polar- and Marine Research maintains the LTER series of the Biologische Anstalt Helgoland, Helgoland Roads in the German Bight (North Sea, 54°11'N 7°54'E), approximately 60 km off the German mainland. The dataset represents the longest most detailed daily dataset available [2]. It comprises a phytoplankton time series (started in 1962) and a zooplankton time series (started in 1975) along with time series for inorganic nutrients, salinity and temperature. More recently, these regular long-term measurements are complemented with automated measurements via a stationary FerryBox System that was installed on Helgoland in 2005. A FerryBox-System is an autonomous or semi-autonomous device located on ships of opportunity or fixed monitoring platforms (as on Helgoland) and has the capacity to autonomously generate information on chlorophyll *a* (Chl*a*) concentration and other key oceanographic parameters [6]. The high sampling frequency of the Helgoland Roads time series has provided a unique opportunity to study long-term trends. This includes abiotic and biotic parameters, but also ecological phenomena such as dynamics and timing of the spring bloom, seasonal interactions between different food web components, niche properties, and the effects of newly introduced species [2;3;7]. The assessment of phytoplankton in the framework of the LTER Helgoland Roads is currently mainly based on microscopic identification and cell counts determined by morphological features of the observed cells. Light microscopy, commonly used for eukaryotic microbial assessment, is very time consuming and requires a high level of taxonomic expertise. Therefore, unlike the Helgoland Roads time series, most phytoplankton time series based on routine microscopy are limited with respect to both temporal and taxonomic resolution. Furthermore, information on occurrence and abundance of smaller phytoplankton species is limited, as different genera with lacking sufficient morphological characteristics for identification are often amalgamated into size classes of different unidentified species. Over the past two decades molecular techniques have become indispensable tools in phytoplankton research, encompassing both evolutionary and ecological studies [8;9;10]. These molecular techniques also hold great promise for overcoming the limitations of phytoplankton time series which rely predominantly on microscopic assessments as described above. Next generation sequencing (NGS) in particular has provided high resolution information on taxonomic composition of marine protist communities [11;12;13]. Decreasing analysis costs in this field coupled with considerable progress towards a greater level of automation into the sampling and analysis process now facilitate integration of molecular based observations into LTERs in order to refine regular observations of marine microbes.

In this study, we used the remote-controlled automated filtration system AUTOFIM (iSiTEC GmbH, Germany) to collect samples of marine microbes for molecular analyses. Measurements of key physicochemical parameters via an adjacent FerryBox-system complemented the sampling. Based on this unique combination of three cutting-edge marine observational approaches we were able to provide novel insights into changes in the eukaryotic microbial community composition towards the end of the spring bloom 2016 for better understanding eukaryotic plankton succession and bloom phenology in the context of the highly variable physicochemical environment at the island of Helgoland.

## Materials and methods

### Study area

**Fig 1:**
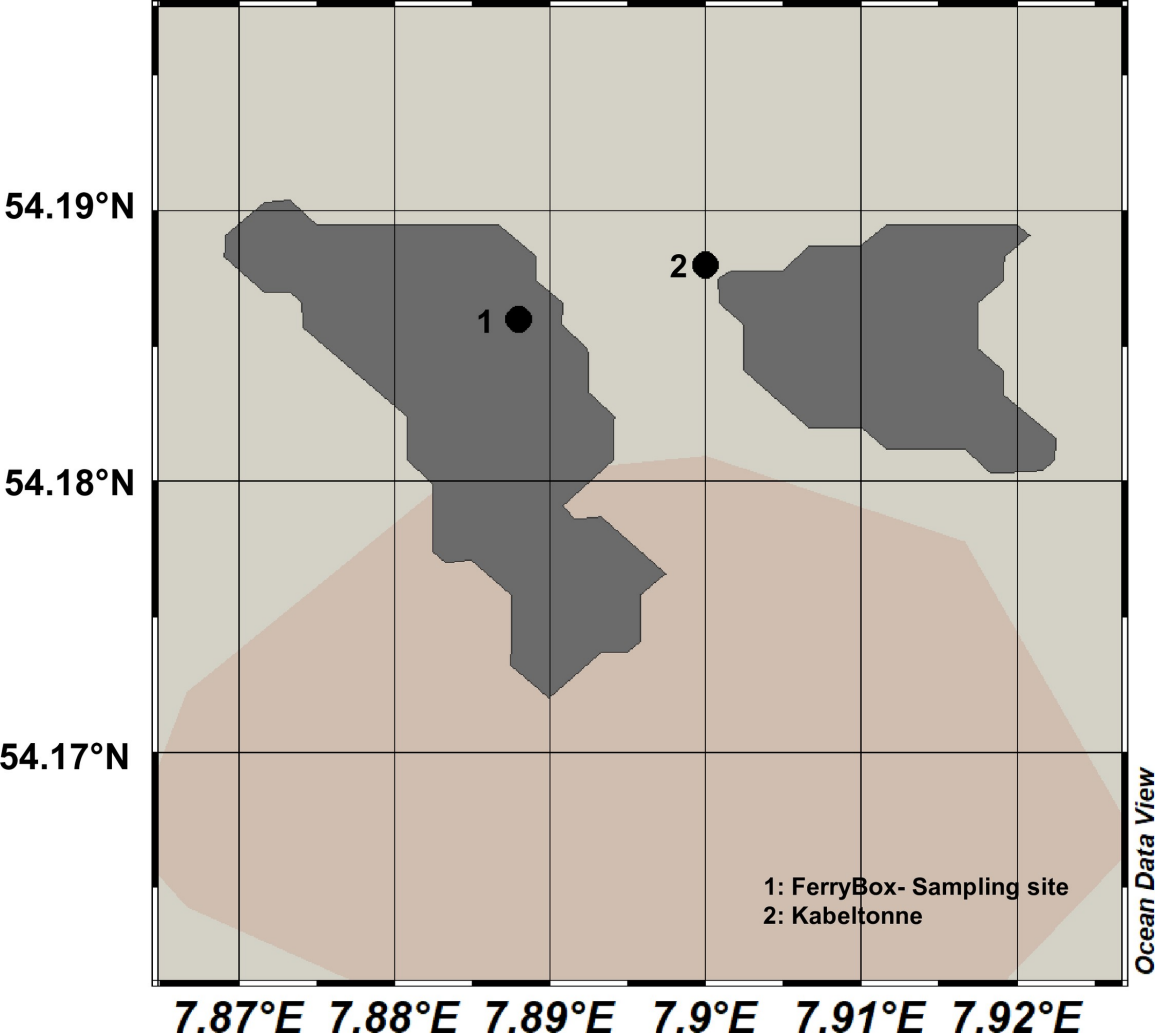
Map of Helgoland with sampling sites.

The AUTOFIM device was installed directly adjacent to a FerryBox System on the island of Helgoland. The sampling site will hereafter be referred to as: “FerryBox-sampling site” (Fig 1). All water samples analyzed via sequencing in this study originated from the same water intake pipe. Physicochemical parameters such as salinity, temperature, and pH were measured in real-time at this site [2].

### Sampling

**Fig 2:**
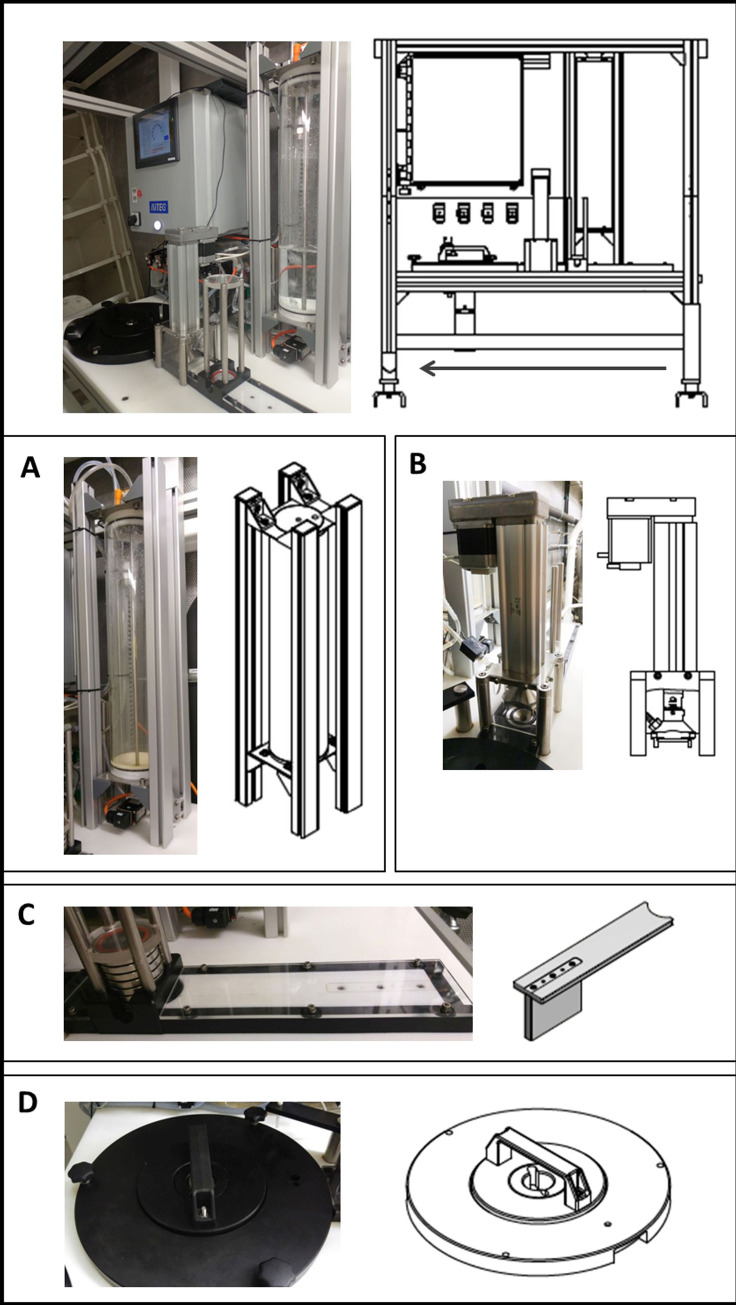
**Photos and drawings of the entire automated filtration system AUTOFIM (upper panel).** The arrow in the upper panel represents the linear working direction of the push bar and the stetting of the treatment units (A-D). A: sample reservoir, B: filtration unit, C: push bar for filter application, D: sample archive.

The sampling sites of this study are operated as part of the LTER Helgoland Roads. This is a time series operated by the Alfred-Wegener Institute. The time series’ principle investigator has given permission to use samples collected as part of this study. Marine microbial samples were collected for molecular analyses via manual filtration and AUTOFIM twice a week in May 2016 (Table 1), resulting in a set of eight samples from each sampling method for subsequent DNA-isolation. AUTOFIM is suited to collection up to 12 samples autonomously. There are three sampling options: (i) remote controlled, (ii) from fixed time-points, (iii) at fixed time-intervals. The system can collect samples with volumes up to five liters of water. The sampling volume can be customized in the range between 0.05–5 liters depending on the sampling strategy and expected cell densities. In case of a clogged filter, filtration is cancelled and the filtered volume is documented. The filtration system mainly consists of three basic units: (i) a sample reservoir for water sampling with customized sample volumes, (ii) a filtration unit and (iii) a sample archive consisting of a sealing disc with individual spaces for storage of the filters (Fig 2). The complete system is being installed on a support frame that allows easy transport and installation either at fixed monitoring sites, e.g. at Helgoland or on board ships (research vessels or on ships of opportunity). The automatic flow control is achieved by an industrial **P**rogrammable **L**ogic **C**ontroller (PLC) which can be provided with random interfaces including Ethernet and Transmission Control Protocol/Internet Protocol. Thus, communication, remote-controlling and data exchange with existing systems (e.g. with the Ferry Box System) and networks including event-controlled sampling is possible.

**Table 1:** Physicochemical parameter at the study sites (KT:”Kabeltonne”; FB:”FerryBox-sampling site).

			Temperature [°C]		Salinity		pH		SiO_4_ [μmol/l]	PO_4_ [μmol/l]	NO_2_ [μmol/l]	NO_3_ [μmol/l]	NH_4_ [μmol/l]	Chl*a* [μg/l]
Period	Week	Date	KT	FB	KT	FB	KT	FB	KT					
**A**	**1**	**May 3**	8.8	9.1	32.8	32.8	n.a.	8.3	0.1	0.1	0.2	7.7	0.1	6.85
		**May 6**	8.2	9.8	32.7	32.8	n.a.	8.3	0.3	0.2	0.1	5.5	0.5	6.53
	**2**	**May 10**	9.8	10.7	32.7	32.8	8.5	8.3	0.4	0.2	0.1	5.1	0.9	7.03
		**May 12**	10.4	10.8	32.8	32.8	8.5	8.3	0.7	0.6	0.1	3.3	2.6	6.83
**B**	**3**	**May 17**	10.4	11.1	31.9	31.9	n.a.	8.4	0.4	0.1	0.1	7.2	1.5	5.25
		**May 19**	10.6	11.7	32.0	32.1	n.a.	8.3	0.4	0.1	0.2	11.1	n.a.	3.20
	**4**	**May 24**	11.5	11.8	31.5	31.5	8.3	8.3	1.4	0.1	0.1	10.2	0.6	3.21
		**May 26**	11.4	11.7	31.7	31.7	8.2	8.3	2.1	0.2	0.2	6.6	0.4	2.12

Prior to sampling, membrane filters had to be placed manually in a flat V4A-filter holder with a frit (Satorius, Germany) and fixed with an O-ringed gasket (Trelleborg Sealing Solutions GmbH, Germany). The filter holders were stored in a vertical stacker in a filter magazine. A push bar was used to slide the filter from the filter magazine in direct position under the filtration unit. Water samples were pumped through the filtration unit to obtain marine microbes or particles for further molecular analysis. Via AUTOFIM we filtered one liter of sea-water onto Isopore Membrane Filters with a diameter of 45 mm and a mesh-size of 0.4 μm (Millipore, USA) at maximum 200 mbar. Any other filter with a diameter of 45 mm may also be used for filtration with the device. Subsequent to the filtration procedure the filter was automatically transported into the preservation position of the sample archive. Here ~2 ml of a preservation buffer (4M Guanidine thiocyanate, 25mM Sodium citrate dehydrate, 0,5% Triton™ X-100, 300mM Sodium chloride, 20mM Trizma® base, 2mM Ethylenediaminetetraacetic acid disodium salt dehydrate, 0,04% Sodium dodecyl sulfate, pH 8.4) was applied to the filter by a dosing unit. The dosing unit consists of a reservoir for preservation solutions and an electrically actuated pump that releases a defined volume of the solution on the filter. Subsequent to filtration, the filter was automatically moved via the push bar into the sample archive. Samples were removed from the sample archive and stored until DNA-extraction in preservation buffer at -20°C in a freezer.

The samples for manual filtration were collected via one liter glass bottles (Schott, Germany). Manual filtration was carried out at maximum 200 mbar using Isopore Membrane Filters (Millipore, USA) with the pore-size 0.4 μm and a diameter of 45 mm. In accordance to standard sampling protocols for environmental DNA (e.g. Metfies et al., 2016), the samples collected via manual filtration were directly frozen at -20°C.

### Microscopic counting

Water samples for microscopic counting were collected at station “Kabeltonne” (Fig 1). This site is between the main island of Helgoland and the island Düne Helgoland to the west (54°11'09.8"N 7°53'16.6"E). This site is located in the same narrow channel, dominated by tidal currents (Hickel 1998), as the “FerryBox-sampling site” (both are roughly 650 m apart). The sites are shallow (<10 m) and due to the strong currents and high degree of turbulence the water column in the area is usually well-mixed and samples are therefore considered representative for the resident water mass at LTER Helgoland Roads [2]. These samples for microscopic counting were preserved with a 0.1% neutral Lugol´s iodine solution following the same methodology as the regular Helgoland Roads sample protocol [2]. Environmental parameters and the samples were analyzed following the Helgoland Roads protocols [2]. Microscopic counts attempted to identify selected taxonomic groupse.g. choanoflagellates, which are not usually identified as taxonomic entities in the Helgoland Roads time series.

### DNA-isolation

Isolation of genomic DNA from the field samples was carried out using the NucleoSpin Plant Kit (Machery-Nagel, Germany) following the manufacturer’s protocol. DNA isolation was not successful for the sample collected via AUTOFIM on May 6^th^. Samples were processed within one to two weeks subsequent to sampling. DNA concentrations were determined using the Quantus Fluorometer (Promega, Germany) according to the manufacturer’s protocol for measuring double stranded DNA. The resulting DNA-extracts were stored at -20°C.

### Illumina-Sequencing 18S rDNA

For Illumina-Sequencing, a fragment of the 18S rDNA containing the hypervariable V4 region was amplified with the primer set 528iF (5’- GCGGTAATTCCAGCTCC-3’) [14] and: 938iR (5’-GGCAAATGCTTTCGC-3’). All PCRs (polymerase chain reaction) had a final volume of 25 μl and contained 12.5 μl mastermix (KAPA HiFi HotStart ReadyMix, KAPABiosystems, Roche), 2.5 μl of each primer (1 μMol) and 2.5 μl genomic DNA (~ 5 ng/μl). PCR amplification was performed in a thermal cycler (Eppendorf, Germany) with an initial denaturation (95°C, 3 min) followed by 25 cycles of denaturation (95°C, 30 sec), annealing (55°C, 30 sec), and extension (72°C, 30 sec) with a single final extension (72°C, 5 min). The PCR products were purified from an agarose gel 1% [w/v] with the AMPure XP PCR purification kit (Beckman Coulter, Ing., USA) according to the manufactuer’s protocol. Subsequent to purification of the 18S rDNA fragment the DNA concentrations of the samples were determined using the Quantus Fluorometer (Promega, USA). Indices and sequencing adapters of the Nextera XT Index Kit (Illumina, USA) were attached via the Index PCR. All PCRs had a final volume of 50 μl and contained 25 μl of KAPA HIFI Mix (Kapa Biosystems, Roche, Germany), 5 μl of each Nextera XT Index Primer [1 μmol/L], 5 μl DNA-template [~5ng] and 10 μl PCR grade water. PCR amplification was performed in a thermal cycler (Eppendorf, Germany) with an initial denaturation (95°C, 3 min) followed by 8 cycles of denaturation (95°C, 30 sec), annealing (55°C, 30 sec), and extension (72°C, 30 sec) with a single final extension (72°C, 5 min). Prior to quantification of the PCR products with the Quantus Fluorometer (Promega, USA) for sequencing with the MiSeq-Sequencer (Illumina, USA), the final library was cleaned up using the AMPure XP PCR purification kit (Beckman Coulter, Ing., USA). Sequencing of the DNA-fragments was carried out with the MiSeq Reagent Kit V3 (2 x 300 bp) according to the manufacturer’s protocol (Illumina, USA). Raw sequences generated in this study have been deposited at the European Nucleotide Archive (ENA) with the accession number PRJEB38665.

### Sequence analyses

Raw reads were quality trimmed with Trimmomatic [15]. Thus, reads were scanned with a 4-base wide sliding window and cut when the average quality dropped below 15. For merging paired-end reads, the script join-paired-ends within the open-source bioinformatics pipeline QIIME v.1.8.0 [16] was used with a minimum read overlap of 20 bases. Further analyses were performed following an pipeline developed in-house using QIIME v.1.8.0 (Caporaso et al. 2010). In short, reads were quality-filtered according to recommended settings [17]. Only sequences that fully matched the primer sequences at the beginning and end of the sequence, respectively, and which were between 200 and 500 bp in length were further processed. For chimera detection and clustering of sequences into OTUs we used the QIIME workflow ‘usearch.qf’, which incorporates UCHIME [18]. Pre-clustered sequences were checked for chimeras (*de novo* and with Silva 119 SSU). The remaining sequence set was clustered (*de novo*) into OTUs with a similarity threshold of 98%. The taxonomy of OTUs were classified using PR^2^ and the QIIME default sequence classifier UCLUST [19]. Normalization, analyses and visualisation of the sequence data was carried out in R (R Development Core Team, 2008). The sequence dataset was normalized to the smallest number of high quality sequences obtained for any sample in this sample set (35.000 sequences) using the rarefy function from the Vegan package. Visualisation and analyses of the 18S amplicon data was based on ampvis2 [20] and the R package Pheatmap.

### Statistical analyses

Statistical analyses were carried out in the R-software (R Development Core Team, 2008). MANTEL tests based on the Pearson correlation coefficient and 999 permutations were carried out on the basis of Euclidian distance matrices calculated for the entire dataset and performed with R package “vegan” in order to assess possible correlations between the environmental data from the FerryBox-sampling site and “Kabeltonne”. The principal component analysis (PCA) and ANOSIM were carried out with the R package “vegan”. Correlations between sequence abundances and microscopic counts were determined using the ggpubr R package taking advantage of the Pearson correlation coefficient.

## Results

### Physicochemical conditions at Helgoland Roads during the period of the molecular survey

The observation period was characterized by a shift in environmental conditions after period A, i.e. between May 12^th^ and 17^th^. The shift was characterized by an increase in temperature and silicate concentration accompanied by a decrease in salinity (Table 1). Chl*a* concentrations at the FerryBox-sampling site as determined via fluorescence measurements were significantly (*p* = 0.01) higher during period A of the observation period (average 6,81 μg/L) than during period B (average 3,45 μg/L). This difference reflects the decline of the phytoplankton bloom. A MANTEL-test revealed that physicochemical conditions (T; S; NO_2_; Chl*a*) at the two sampling sites FerryBox-intake and Kabeltonne were highly similar (*R* = 0.93 and *p* = 0.001) during the observation period. This suggests that samples collected at “Kabeltonne” and the “FerryBox- sampling site” originated from the same water mass and justifies a direct comparison of the microscopic count data from “Kabeltonne” with molecular data obtained from the FerryBox-intake later on in this study.

### Illumina sequencing

**Fig 3:**
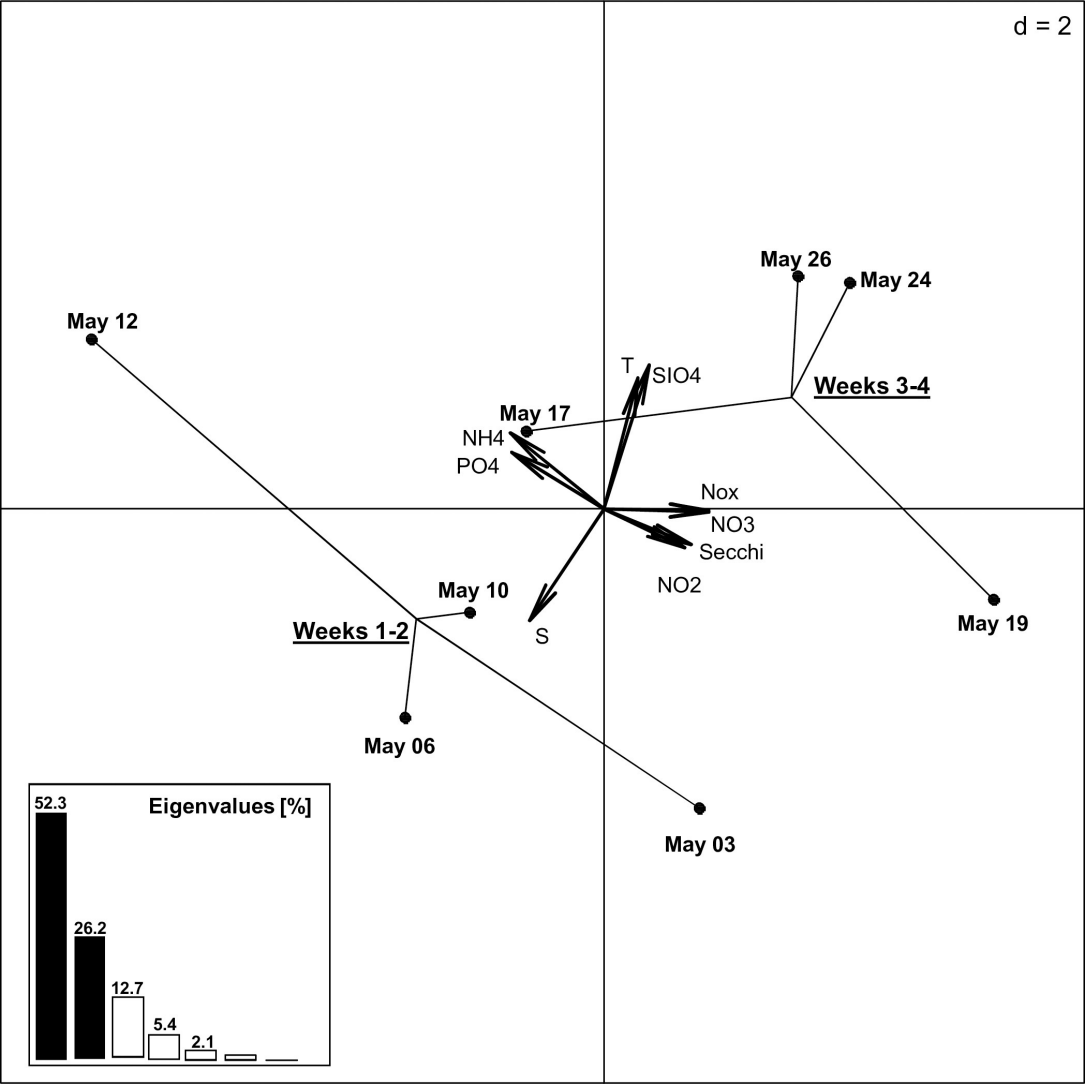
Principle Component Analyses (PCA) illustrating the impact of a shift in environmental conditions on the community composition during the observation period.

After quality-filtering the Illumina sequencing generated 1.239.033 high quality reads of the 18S rDNA V4-region. Individual samples contained 64.434–103.020 sequences (mean 82.602 sequences per sample) resulting in 8150 different OTUs (mean 1417 OTUs per sample). Post normalization to 35.000 sequences per sample and exclusion of OTUs with overall sequence abundance <0.05% the remaining sequences clustered into a total of 170 different OTUs. Individual samples contained 71–153 OTUs (mean 136 OTUs per sample) that represented 80–91% (mean 87%) of the reads obtained from the individual samples (Table 2). The OTUs represented all major taxonomic groups expected to be found in eukaryotic microbial plankton samples in the vicinity of Helgoland Roads such as dinoflagellata, haptophyta, chlorophyta, ciliophora, bacillariophyta (ochrophyta), choanoflagellida, and oomycota. [21;22;23;24]. The communities retrieved from samples collected with AUTOFIM segregate into two major groups reflecting period A and B of the observation period, as characterized by shifts in salinity, temperature and silicate concentration (Fig 3).

**Table 2:** Sequencing statistics.

Sample	Date	High Quality Reads	OTUs from High Quality Reads	OTUs Post Normalisation	Shared OTUs (A&M) [%]	OTUs > 0.05% Post Normalisation	Reads >0.05% Post Normalization [%]	Shared OTUs > 0.05% (A&M) [%]
**AUTOFIM (A)**								
HLG_030516_A	May 03	64434	1419	1108	88	143	87	92
HLG_100516_A	May 10	80321	1594	1161	85	148	87	93
HLG_120516_A	May 12	88506	1713	1280	85	146	84	94
HLG_170516_A	May 17	64894	1413	1087	87	144	90	94
HLG_190516_A	May 19	88234	1554	1048	88	144	88	89
HLG_240516_A	May 24	102552	731	596	88	95	80	65
HLG_260516_A	May 26	103020	1598	1044	88	136	89	88
**Manual Fitration (M)**								
HLG_030516_M	May 03	84508	1388	978		142	89	
HLG_060516_M	May 06	71411	1607	1194		150	85	
HLG_100516_M	May 10	69374	1573	1280		153	84	
HLG_120516_M	May 12	72239	1677	1244		150	86	
HLG_170516_M	May 17	92852	1479	973		140	91	
HLG_190516_M	May 19	91379	1560	1057		143	90	
HLG_240516_M	May 24	98624	646	581		71	86	
HLG_260516_M	May 26	66685	1300	1034		140	86	
**Mean**		**82602**	**1417**	**1043**	**87**	**136**	**87**	**88**

### Evaluation automated filtration on molecular analyses

**Fig 4:**
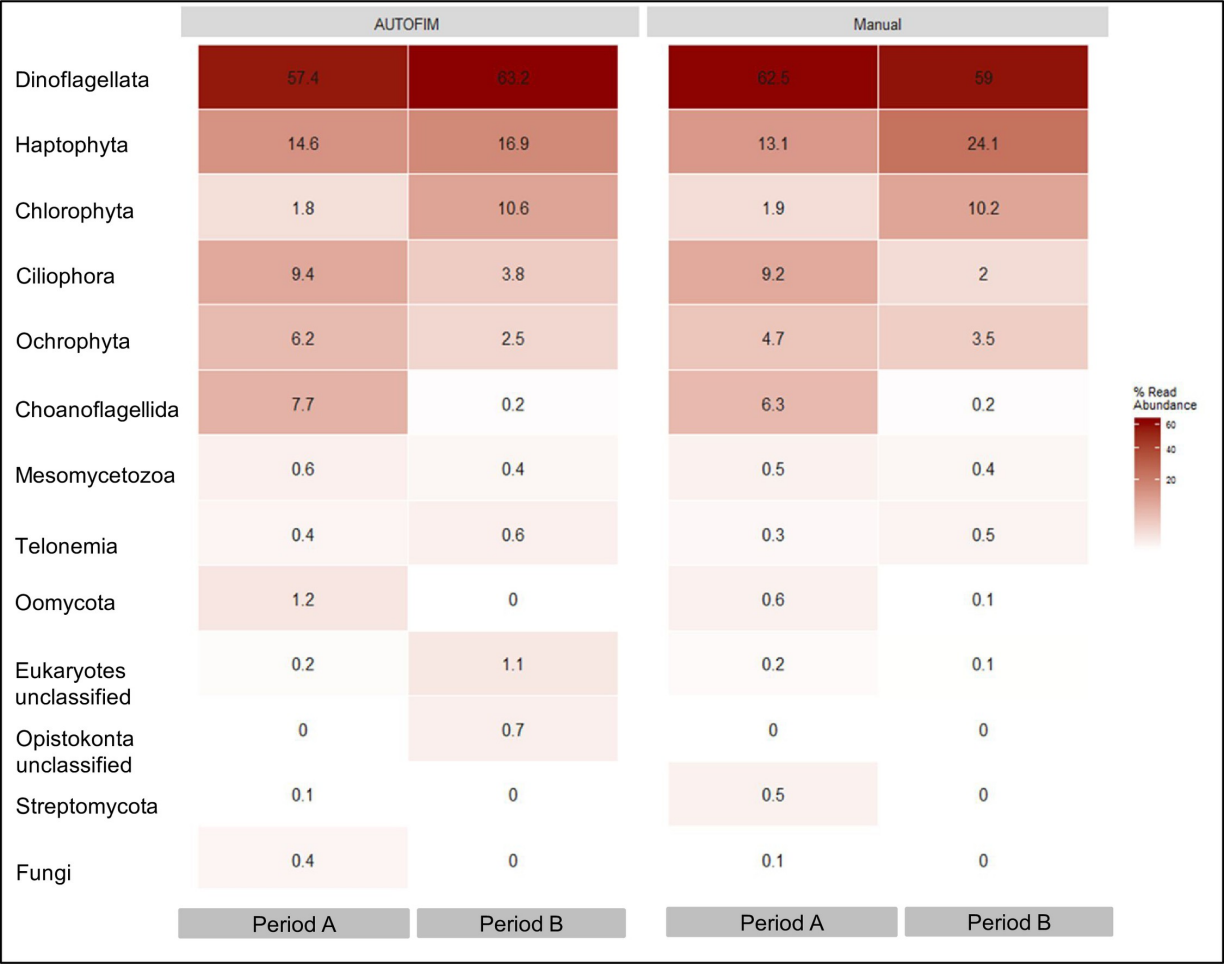
Mean relative sequence abundances retrieved from samples collected with AUTOFIM, respectively manual filtration during period A and period B of the observation period.

The impact of automated filtration and preservation on the results of a molecular assessment of plankton community composition was evaluated by comparing sequence libraries obtained from samples collected at the FerryBox-sampling site via AUTOFIM with samples collected via manual filtration. The average number of OTUs retrieved from a sample was highly similar for both two approaches. Prior to exclusion of OTUs with overall sequence abundance <0.05% on average 1434 OTUs were obtained from the automated filtration, while 1404 OTUs were obtained from samples collected via manual filtration. Subsequent to exclusion of OTUs with low sequence abundance 136 OTUs were obtained from both sampling approaches (Table 2). Furthermore, the two methods were in agreement on the presence or absence of an OTU for ~ 90% of the OTUs (Table 2) and relative sequence abundance of the higher taxonomic groups (Fig 4). Community compositions inferred from preserved samples collected with AUTOFIM did not significantly differ from community compositions from samples collected manually and directly frozen (ANOSIM; R = -0.11; p = 0.97).

### Community structure and succession of marine eukaryotic microbes during the observation period

**Fig 5:**
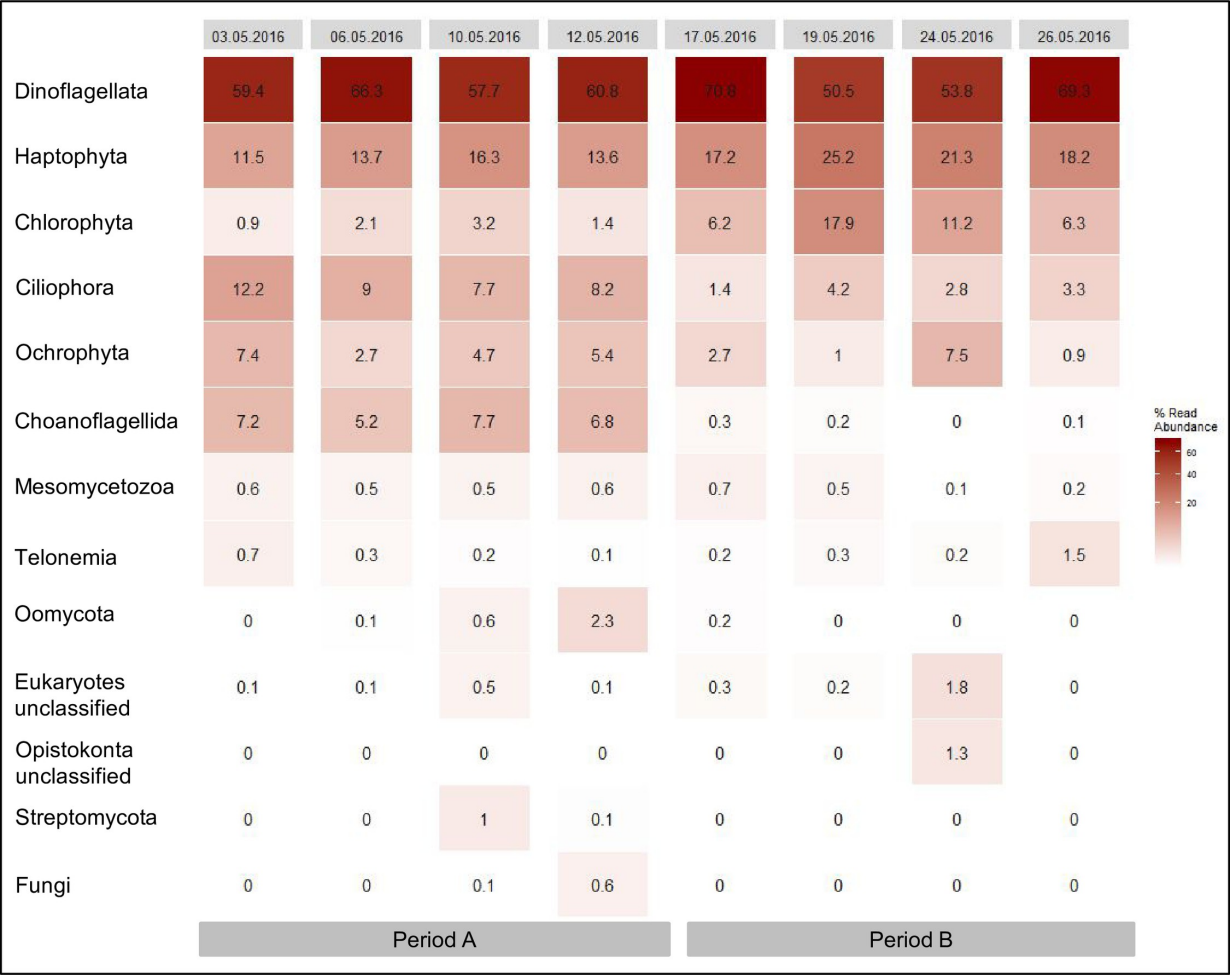
Relative sequence abundances by sampling days retrieved from samples collected with AUTOFIM.

Dinoflagellata, haptophyte, chlorophyte, ciliophora, ochrophyta, and choanoflagellida constituted more than 90% of all eukaryotic microbial sequences identified in the individual samples (Fig 5). The community changed significantly from period A to period B. Sequence abundances of ochrophyta, ciliophora and choanoflagellida were higher in period A than in period B, while chlorophyta and haptophyta sequence abundances were lower in period A than in period B (Fig 5).

**Fig 6:**
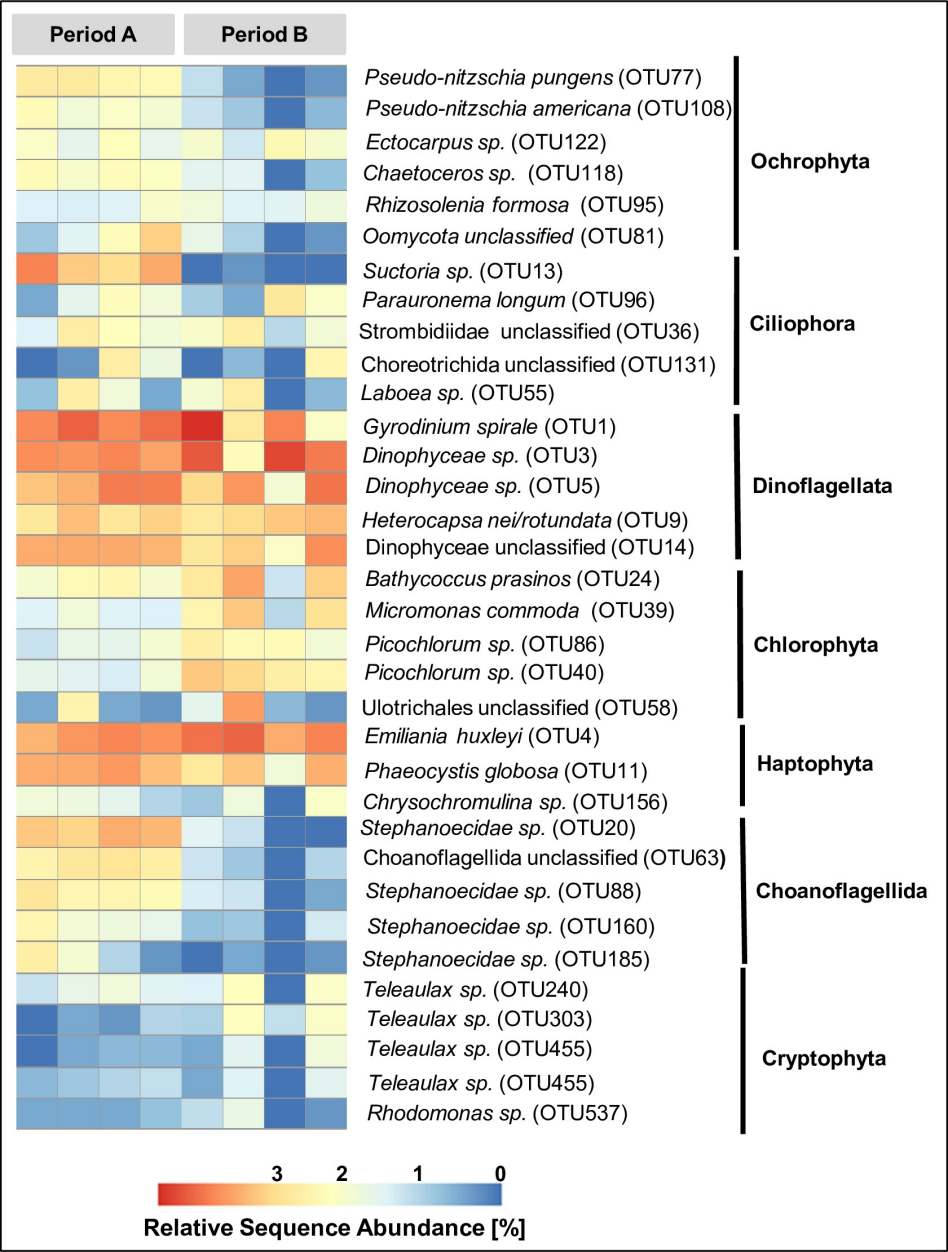
Relative sequence abundance of the taxa dominating the respective higher taxonomic group.

A decline in sequences of the larger phototrophic diatom genera *Pseudo-nitzschia*, and *Chaetoceros* specifically characterized the decline in ochrophyta sequences (Fig 6). An OTU with 99% similarity to the diatom parasite *Miracula helgolandica* increased during period A and peaked in abundance on May 12^th^. Subsequent to the peak in sequence abundance of *Miracula helgolandica*, sequence abundance of *Pseudo-nitzschia pungens* was significantly reduced, suggesting a potentially negative impact of the parasite on the growth rate of this diatom species.

Ciliophora showed a similar trend in relative sequence abundance as stramenopiles during the observation period. Relative sequence abundance of ciliophora was higher in period A than in period B. Sequence assemblages of ciliophora were best represented throughout period A by sequences affiliating with the subclass suctoria (Fig 6). The relative sequence abundance of this taxon decreased from period A to period B. Choanoflagellates (holozoa) also significantly declined in relative sequence abundance from period A to period B. In our dataset the decline of choanoflagellates was best represented by a decline of relative sequence abundance of *Stephanoeca* (Fig 6). Chlorophyta and haptophyta are two taxonomic groups recognized in this dataset whose sequence abundance significantly increased from period A to period B. Chlorophyta were mainly represented in this dataset by sequences that are affiliated with the pico-eukaryote genera *Bathycoccus prasinos* and *Micromonas commoda* (Fig 6). Haptophyta were best represented in the dataset by sequences affiliating with *Emiliania huxleyi* and *Phaeocystis globosa* (Fig 6). However, relative contribution of the two taxa developed differently over the observation period. *Emiliania huxleyi* increased in relative sequence abundance from period A to period B, while *Phaeocystis globosa* decreased at the same time. The relative sequence abundance of the dinoflagellata were stable throughout the observation period. Among the dinoflagellata 18S rDNA sequences of *Gyrodinium spirale* (Gymnodiniphycidae) had the highest relative sequence abundance in the dataset. In the framework of LTER Helgoland Roads assessment of Cryptophyceae is restricted to global enumeration of cells that belong to this taxonomic group. In this study sequencing data provide an insight into the taxonomic composition within this group during the observation period. The genera *Teleaulax*, and *Rhodomonas*, constituted the Cryptophyceae population during the observation period. *Teleaulax* increased in relative sequence abundance from period A to period B, while *Rhodomonas* did not show a change in relative sequence abundance. In addition to 18S rRNA gene sequences of eukaryotic microbes we found sequences of the macroalgal taxa *Ulva* and *Ectocarpus* that did not display a change in relative sequence abundance during the observation period. With the exception of cryptophyta the 18S meta-barcoding based information on changes in eukaryotic microbial community composition at Helgoland was confirmed by microscopic data, that correlated well with the molecular based information for *Chaetoceros* (R = 0.83; *p* = 0.01), coccolithophores (R = 0.59; *p* = 0.12), *Phaeocystis* (R = 0.67; *p* = 0.07), and choanoflagellates (R = 0.85; *p* = 0.01) (Table 3).

**Table 3:** Microscopic counts and sequence abundances of selected taxonomic groups. Seq.: mean value for sequence abundance from AUTOFIM and manual filtration.

	03.05	06.05	10.05	12.05	17.05	19.05	24.05	26.05	R	p
Seq. *Chaetoceros sp*.	121	76	121	100	37	61	2	10	0.83	0.01
Counts *Chaetoceros sp*. [cells/ml]	1620	598	735	872	182	23	69	92		
Seq. Cryptophyta	15	10	28	17	26	122	4	71	0.26	0.54
Counts Cryptophyta [cells/ml]	56	83	55	120	374	289	563	576		
Seq. Coccolithophores	909	1574	2022	1689	2949	3593	1053	2231	0.59	0.12
Counts Coccolithophores [cells/ml]	96	125	69	188	1301	781	709	338		
Seq. *Phaeocystis sp*.	65	51	89	71	22	0	1,36	3	0.67	0.07
Counts *Phaeocystis sp*. [cells/ml]	1095	1134	1571	792	278	709	71	1041		
Seq. Choanoflagellates	1420	1025	1572	1210	57	37	0	26	0.85	0.01
Counts Choanoflagellates [cells/ml]	335	175	152	110	14	16	2	1		

## Discussion

Many ongoing marine time series including Helgoland Roads LTER have provided clear evidence for the potential impacts of climate change on phytoplankton communities and phytoplankton bloom dynamics [25;26;27]. Nevertheless, due to current sampling and analysis constraints, such community effects are being judged on the basis of an incomplete picture, as considerable proportions of the eukaryotic microbial network are not regularly enumerated or sampled in many time series. It can therefore be expected, that the scientific value of these regular marine plankton observation programs could further be increased if current limitations with respect to an even higher resolution sampling at spatial and temporal scales than the current daily sampling. Especially the taxonomic resolution of the assessments, and parallel availability of key physicochemical parameter may enhance time series observations. In this study, we demonstrate that the integration of the AUTOFIM filtration system with a FerryBox-System and subsequent 18S meta-barcoding could be a significant step forward to overcoming the taxonomic limitations of the regular marine plankton observation programs outlined above. Based on this approach, we assessed the eukaryotic microbial community composition at Helgoland throughout the entire month of May 2016, to gain more detailed insights into the dynamics of the plankton community at the end of a bloom. We included the locally little investigated component of phytoplankton parasites, and heterotrophic and autotrophic nanoflagellates and found them to play a role in the succession of the eukaryotic community.

The installation of the newly developed automated filtration device AUTOFIM adjacent to a FerryBox at the FerryBox-Sampling Site on Helgoland complemented the taxonomic information with near real time information on the physicochemical conditions at the time of the sampling. AUTOFIM can be deployed either as a stand-alone system or as an additional module of the FerryBox-System at fixed monitoring sites such as Helgoland Roads, but also on moving platforms such as research vessels [28] or ships of opportunity. Over the past decade publications have demonstrated the power of the FerryBox system to provide high resolution real-time information on key physicochemical parameters [6;29;30], and the suitability of molecular methods for observation of marine plankton diversity [23;31;32;33;34]. Sampling volumes reported in published molecular studies of eukaryotic marine microbial biodiversity range from less than hundred milliliters to hundreds of liters [35], while conventional light microscopic surveys of marine plankton composition are usually based on settling 25–50 mL of Lugol´s-fixed water subsamples for cell counting. A recent study comparing molecular based data with light microscopic counts revealed higher correlations between the two approaches for smaller taxa than for larger taxa. It was hypothesised that this was due to the small sample volumes of less than 100 ml usually used for microscopic analysis, which is a factor of 10–20 smaller than sample volumes taken for molecular analyses [36]. These findings suggest, that the volumes routinely analysed in microscopic analyses are not very well suited to generate a representative picture of plankton community composition, while another study reports, that sampling volumes to detect 90% of phytoplankton range from 0.25–1 liter depending on the concentration of phytoplankton in a given sample [37]. At Helgoland Roads the minimum cell number cut off point for counts is four cells and a size below approximately 5 μm, taxonomic identification of some organisms becomes difficult. The flexibility of AUTOFIM to customize the sampling volume in a range between 0.05–5 liters is a valuable feature to optimize sampling volumes for marine eukaryotic microbial community analyses depending on the underlying research question. In the last decade other technical solutions for *in situ* microbial sample collection have been published for different state of the art genetic assays for microbial and biogeochemical downstream sensing and lab-based analytics, as reviewed by [38]. To date a number of different ocean-deployable microbiological sampling tools are available, which collect water samples and/or particulate organic matter *in situ*. However, to our knowledge, most of these autonomous sampling devices e.g. the automated water sampler (WaMS, [35]), the Remote Access Sampler (RAS, MacLane laboratories), the Water Sampler for underwater Autonomous Vehicles [39], and the Environmental Sample Processor (ESP) collect water samples with a non-adjustable volume of 500 ml or less [40]. Based on these sampling devices it is difficult to provide larger sample volumes, if needed.

Long time-periods between sample collection and return to lab for processing and downstream applications increase the risk of changing physicochemical conditions and decomposition processes in the sample, which can introduce artefacts. At Helgoland Roads short times back to the lab assure that short processing times for analyses. However, at sites far from the lab, this alteration is minimized by *in situ* fixation of water samples directly subsequent to the sample collection. Cells and the integrity of sensitive molecules (e.g. nucleic acids) are archived by addition of commercial or custom-made preservatives [41]. Several instruments are designed with integrated sampling and preservation tools, e.g. the ESP. Preservation of ESP samples with a nucleic acid preservative yielded high quality nucleic acids even after 30 days of incubation at room temperature or on-board the sampling device, demonstrating the stability of nucleic acids subsequent to application of an adequate preservative [42]. In this study we obtained similar results using a custom-made Guanidine thiocyanate-containing preservative to avoid sample bias introduced by degradation processes and demonstrate that application of our custom-made preservative in combination with automated filtration does not significantly bias the molecular based biodiversity information obtained from the samples. This was also observed in a previous study for other liquid nucleic acid preservatives such as DNAgard (Biomatrica, USA) or RNAlater (Ambion, USA) [43]. However, high volumes of preservative are often required using other sampling tools, increasing the commercial cost of the deployment. For example, 14 samples collected with the Suspended Particulate Rosette (SPR) require around 10 L of RNA*later* for preservation [44]. On the other hand, a 12 microbial sample set collected with AUTOFIM is preserved with only ~24 mL of the preservative used in this study.

The results of our 18S rDNA sequencing identified a total of 170 OTUs with sequence abundance >0.05%. The molecular survey confirmed a high number of individual species or genera in all the larger taxon groups routinely reported at Helgoland Roads (diatoms, dinoflagellates, chlorophytes, coccolithophores and unidentified flagellates) [21;22;23;24]. The data revealed changes in the eukaryotic microbial community after May 12^th^. This is reflected by a change from larger photo- and heterotrophs (diatoms, ciliates) to smaller phototrophs (chlorophytes). The decline of stramenopile (ochrophyta) sequences, such as diatoms and the increase in relative sequence abundance of pico-eukaryotes, like *Micromonas* or *Bathycoccus* indicate that the observation period might have fallen in the termination phase of the diatom spring bloom 2016. This is also reflected by an increase in nutrients such as nitrate and silicate in period two (week 3 and 4 respectively) that are consumed by diatoms during the spring bloom and become re-mineralized in the senescent bloom [45]. During the first half of the observation period, the diatom assemblage was dominated by taxa that are well known to occur during the spring bloom in the water around Helgoland such as *Pseudo-nitzschia* and *Chaetoceros* [46]. However, sequence abundances of these three taxa and other taxa significantly declined throughout the observation period. Very interestingly the decline phase of diatom sequences succeeded a peak in Perenosporales sequences (oomycetes) on May 12^th^ and was accompanied by a decline of ciliate sequences. The decay of ciliates alongside with diatoms might be explained by previous studies that report ciliates to prey on diatoms and respond quickly to food availability [47]. Oomycetes are a taxonomic group for which we only have little quantitative and ecological information at Helgoland Roads, although we know that they occur there [48]. This is partly due to the low incidence of these organisms and different life cycle stages of these organisms, some of which are very inconspicuous and cannot be resolved in routine microscope counts. There are a number of oomycete taxa that are diatom parasites and a considerable number of species of other different taxonomic groups, such as the flagellates *Cryothecomonas aestivalis* (infecting *Guinardia delicatula*), *Phagomyxa odontellae* (infecting *Odontella sinensis* [49] and many others are known to infect diatoms, but their detailed dynamics are rarely quantified [50]. The oomycete *Miracula helgolandica* is known to infect the diatom species *Pseudo-nitzschia pungens* [51], but incidences of parasitic infection of diatoms are often assumed to be relatively low. With current microscopy techniques fungal, oomycete and other parasites can easily be overlooked, but when they occur, their consequences can be significant, essentially by ending a bloom and impacting the entire planktonic food web at that time [52]. In this study sequence abundance of *Pseudo-nitzschia pungens* significantly declined subsequent to a peak in sequence abundance of *Miracula helgolandica*, suggesting that this oomycete parasite might be involved in terminating the diatom bloom. These data demonstrate that molecular monitoring can help to remedy the lack of resolution evinced by light microscopy as it facilitates detection of the target organisms even at low abundances and irrespective of life cycle stage (for instance small zoospores). In this study, the sequence-based analyses also provided information on the occurrence of different Cryptophyceae genera, e.g. *Teleaulax* that significantly contributed to the Cryptophyceae community during the observation period. A previous molecular survey focusing on Cryptophyceae abundance in the German Bight over three annual cycles suggests, that *Teleaulax* is among the taxa that dominate Cryptophyceae community composition at the Helgoland Roads sampling site [23; 32]. The previous data and the meta-barcoding data of this study provide information on the variability in Cryptophyceae community composition at LTER Helgoland Roads. In the framework of other long-term observation time series surveillance of Cryptophyceae is often restricted to enumeration of different size classes based on microscopic counts. Quantifying Cryptophycae below class level, e.g. at genus level can be very time consuming due to uninformative morphologies [53], or when the abundance of these species is very low. Other taxa such as choanoflagellates are identifiable by light microscopy but often disintegrate in fixed samples, as choanoflagellates are prone to lose their outer silica structures. However, in this study our sequence-based analyses provided genus specific information on the relative abundance of choanoflagellates during the observation period and this was significantly correlated with microscopic counts of this group. In summary, particularly for small and delicate taxa, NGS based surveillance of marine eukaryotic microbes could have a considerable added value for time series observations.

## Conclusions

Based on our integrated approach we provided novel insights into the eukaryotic microbial community composition and dynamics at Helgoland Roads towards the end of the spring bloom in May 2016. We observed a shift from larger to smaller phototropic eukaryotic microbes. This included a decline in diatom sequences subsequent to a peak in oomycete sequence abundance suggesting that parasitism can form an important ecological determinant of diatom bloom dynamics. Here, parasites like oomycetes might be of similar importance to grazing, senescence or changing environmental conditions, yet parasitism is rarely considered as a factor at least in marine plankton ecology. Our findings demonstrate that the integration of remote-controlled filtration systems like AUTOFIM in combination with a FerryBox-system and subsequent 18S rDNA meta-barcoding analyses of eukaryotic microbial communities could be a significant step forward augmenting conventional microscopy based observations. It would be very helpful with information on the occurrence of so far understudied taxa. It could help to overcome limitations of sampling in marine plankton observation programs allowing increased spatial- and temporal scales, and higher taxonomic resolution. Technical progress and decreasing analysis costs make it feasible, to jointly apply these techniques on a routine basis for the analyses of marine eukaryotic microbial communities in order to refine information on eukaryotic microbial biodiversity and dynamics in marine long-term observation programs.
